# Use of the Lorenz curve and the Gini index for the evaluation of HIV viral load distribution in an Italian community

**DOI:** 10.3389/fpubh.2025.1580633

**Published:** 2025-07-16

**Authors:** Stefania Arsuffi, Martina Salvi, Stefano Calza, Fabio Riccardo Colombo, Maria Alberti, Benedetta Fioretti, Giorgio Tiecco, Emanuele Focà, Eugenia Quiros-Roldan

**Affiliations:** ^1^Unit of Infectious Diseases, University of Brescia and Spedali Civili Hospital, Brescia, Italy; ^2^Unit of Biostatistics and Bioinformatics, Department of Molecular and Translational Medicine, University of Brescia, Brescia, Italy

**Keywords:** HIV, community viral load, inequality, Lorenz curve, Gini index

## Abstract

**Background:**

Community viral load (CVL) is defined as an aggregate measure of individual viral loads of people living with HIV who are receiving care in a specific community. It serves as a metric to evaluate the effectiveness of therapy programs. Our study aimed to analyze the overtime trend and the distribution characteristics of viral load.

**Methods:**

Using the Lorenz curve, we investigated the demographic characteristics of individuals who comprised the top 10% of viral loads over the years. We applied the Gini index to measure the inequality in the distribution of viral load among the study population.

**Results:**

Our data showed a progressive decrease in community viral load over the years, with a sharp decline after 2012 and a rebound in 2020. The Gini index had a specular trend during this period. In all the observed periods, the top 10% of viral loads showed a progressive increase in the proportion of female individuals and non-Italian nationals.

**Conclusion:**

Our study highlighted the effectiveness and the improvement of the HIV care cascade over the years, with a decrease in community HIV viral load. The introduction of integrase inhibitors seemed to cause a rapid drop in community viral load in 2012 but with a notable discrepancy in the homogeneity of the population. It is also important to highlight the changes over time in the population representing the top 10% of viral loads, with a gradual but stable increase in the proportion of female and non-Italian individuals.

## Background

Community viral load (CVL) is defined as “an aggregate biological measure of viral load for a specific geographic location or community” (1). It is an indicator of HIV transmission potential and the quality of HIV care and treatment in a geographic area, and it may also be utilized for monitoring the progress of HIV care and treatment objectives when assessed over time. It is typically calculated using the arithmetic or geometric mean, median, or sum of the highest or most recent viral loads of all reported HIV-infected individuals with available viral load measurements within a given population over a specified period (2). For CVL to serve as an effective measure, it must accurately represent the viral loads of individuals diagnosed with HIV, be a reliable indicator sensitive to changes in viral load within the community, and reflect sexual behaviors or other transmission risks in the area. It has been stated that local HIV reporting authorities can utilize this metric annually to track progress in administering antiretroviral therapy to HIV-infected individuals. This approach could help lower the community’s overall viral load and potentially reduce the transmission of HIV. Moreover, CVL could be a valuable tool for assessing national HIV care and treatment goals over time (3).

CVL can be stratified by demographics (e.g., age and sex) or epidemiological factors (e.g., risk factors) to identify populations with a higher potential for transmission.

The Lorenz curve (4) was originally developed to represent inequality in wealth distribution and has since been frequently applied in epidemiology and public health to illustrate the distribution of healthcare resources and disease risks (5).

The Gini index and Lorenz curve have also been used to quantify the distribution of viremia among individuals living with HIV to provide valuable insights into the concentration of viral load within a population and, consequently, into the effectiveness of ART. The Lorenz curve approach was applied by Christopoulos et al. (6) to understand the distribution of viral load among people living with HIV in San Francisco. Their results showed that the Lorenz curve offers an innovative way to summarize the distribution of the virus within a population. Specifically, it revealed that nearly all of the viral load (94%) was concentrated within just 10% of individuals—a level of disparity not captured by conventional methods that simply classify viral loads as suppressed or non-suppressed.

In addition, the Gini index was employed to quantify these disparities in cumulative viremia. A higher Gini coefficient indicated greater inequality in viral load distribution, signifying that a small proportion of the population carries a disproportionately high viral load (7). This metric proves useful in identifying subpopulations at a higher risk of transmission, thereby informing targeted interventions to enhance ART uptake and effectiveness.

Further extending this methodology, a study on the epidemiology of HIV population viral load in 12 sub-Saharan African countries utilized both the Gini index and Lorenz curve to assess the distribution of viral load across these diverse settings. This research demonstrated significant variability in viral load distribution, underscoring the importance of equitable access to ART and robust healthcare infrastructure to achieve viral suppression across different populations. The study revealed that countries with lower Gini coefficients generally had better ART coverage and reduced HIV transmission rates, reinforcing the utility of these measures in public health planning and policymaking (8). These studies underscore the utility of the Gini index and Lorenz curve beyond their conventional economic applications.

Our study aimed to analyze the trend over time and the distribution characteristics of viral load among PLWH at the Department of Infectious and Tropical Diseases of Brescia, which is a large outpatient clinic in Northern Italy. We used the Lorenz curve and the Gini index for this analysis.

## Materials and methods

### Study population

This was a retrospective longitudinal single-cohort study conducted at the outpatient HIV clinic, Department of Infectious and Tropical Diseases, ASST Spedali Civili General Hospital, Brescia, Northern Italy, from 1^st^ January 1997 to 31st December 2023. Spedali Civili General Hospital in Brescia is one of the largest public hospitals in Italy and serves as the reference hospital for the local School of Medicine, University of Brescia (University Hospital). It includes the only tertiary referral center for infectious diseases and HIV in the Brescia province, serving approximately 1,300,000 residents, of whom over 4,200 PLWH are currently in follow-up at our center.

All data were gathered from clinical documentation, and no exclusion criteria were applied. The collected data included, among others, age, sex, nationality, sexual behavior, and HIV viremia.

We described community viral load over the years and examined its correlation with demographic characteristics.

Moreover, we divided the observed time span into five-year periods. For each period, we selected individuals within the top 10% of HIV viral load, comparing their demographic characteristics with those of the remaining individuals.

### Microbiological analysis

HIV viremia is routinely used to monitor the response to ART. The frequency of viral load assessments has varied over time in accordance with evolving clinical guidelines and individual patient needs. Specifically, the recommended monitoring interval shifted from approximately every 3 months in the late 1990s to every 4 months in the mid-2000s and to every 6 months since 2015 for patients who are virologically suppressed and clinically stable. Quantification of HIV viral load was performed using branched-DNA assays (up to 2009) and real-time (RT)-PCR from 2010 to 2023.

### Statistical analysis

The Lorenz curve is a graphical representation of the cumulative distribution of a variable—in this case, viral load—across a population. On the x-axis, it plots the cumulative percentage of individuals (from lowest to highest viral burden), and on the y-axis, the cumulative percentage of the total viral load accounted for by those individuals. A perfectly equal distribution of viral load, where every individual carries an equal share, would result in a diagonal line at 45°, known as the line of equality. The greater the curvature of the Lorenz curve away from this line, the higher the inequality in viral load distribution. The Gini index (or Gini coefficient) quantifies this inequality as a single value between 0 and 1 (or equivalently, between 0 and 100%). It is calculated as the ratio of the area between the Lorenz curve and the line of equality to the total area under the line of equality. A Gini index of 0 indicates complete equality—everyone has the same viral load—whereas a Gini index of 1 (or 100%) reflects maximal inequality, where a single individual carries all of the viral burden. In the context of HIV, a higher Gini index implies that a small proportion of individuals carry the majority of circulating virus in the population, which may have implications for transmission dynamics and public health interventions.

In our analysis, HIV viremia was quantified in terms of RNA copies and transformed to the log₁₀ scale. Viral exposure for each individual was measured as copy-years, representing the integral of virus load over the time of exposure. Since viremic load is evaluated at discrete time intervals in practice, the integral was approximated using the trapezoidal rule (9). Trends in proportion changes over time were tested using a chi-squared test for trend in proportions. All statistical tests were performed assuming a two-sided 5% significance level. HIV counts at the patient level were modeled using a generalized linear mixed model (GLMM) with random intercepts to account for the longitudinal data structure (multiple measurements for each patient). A negative binomial family (logarithm link) was used to account for zero inflation in the count data. The results were reported as estimates with corresponding 95% confidence intervals. Following previous studies (6), we divided our population into two groups, with one representing the top 10% and the other the bottom 90% of the viral load distribution. We estimated the association between sexual behavior, sex, and nationality and the probability of belonging to the top 10% of viral load values. Intraclass correlation coefficients (ICCs) were calculated to assess within-group clustering effects. All calculations were performed using the R programming language (version 4.4.1).

### Ethical disclosure

Data were gathered from the clinical documentation of individuals enrolled in the MASTER cohort as aggregate, pseudo-anonymized data. Therefore, approval by the local Ethics Committee was not necessary for this study.

## Results

### Study population

Our population included all PLWH in care from January 1997 (2,494 patients in care) to December 2023 (4,202 patients in care), encompassing both treatment-experienced and treatment-naive individuals. Throughout the entire period, the majority of the participants were male (ranging between 63 and 74% over time), heterosexual (between 59 and 78%), and Italian nationals (ranging between 82 and 95%).

**Figure 1:**
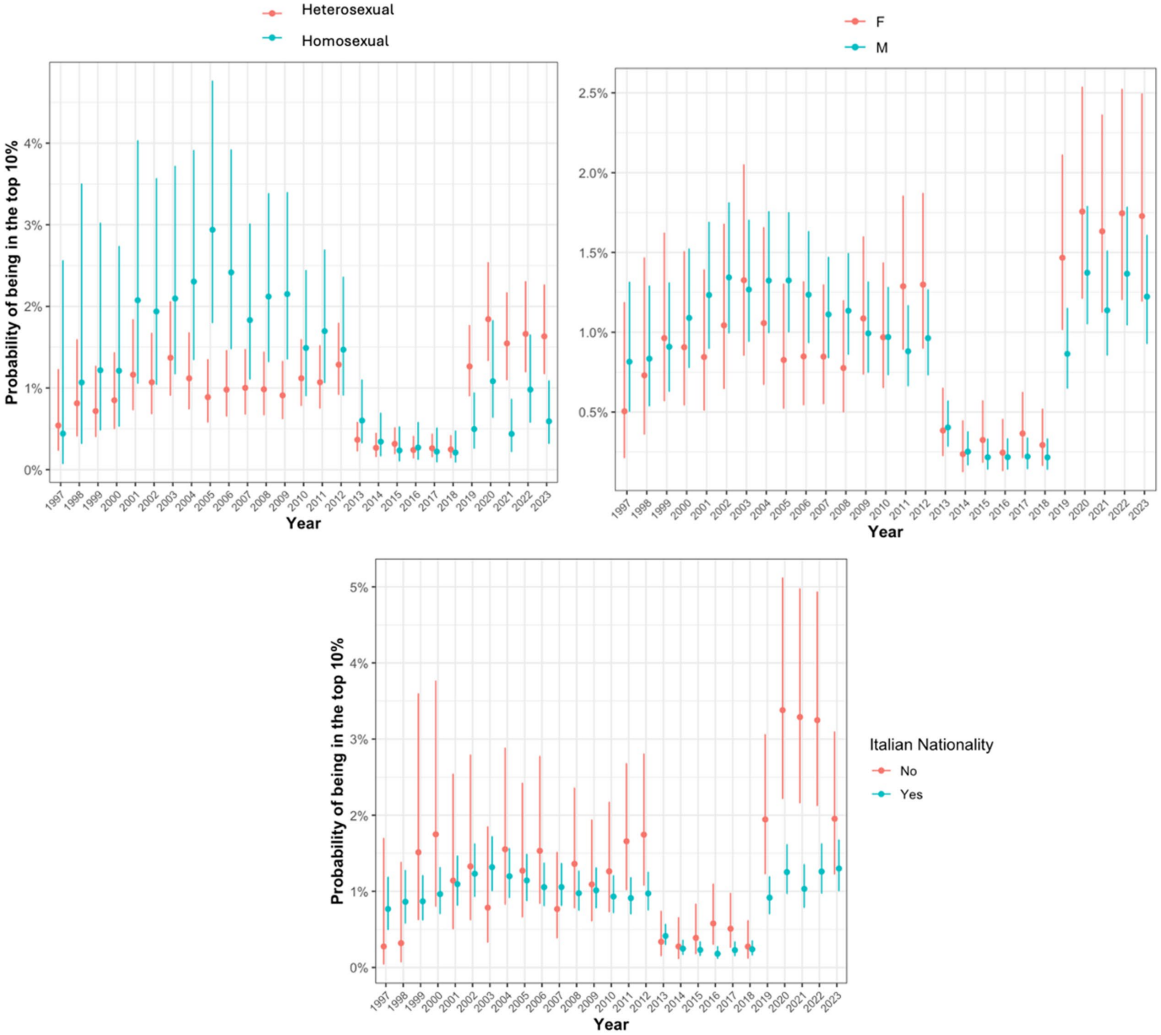
Probability of being in the top 10% viral load group stratified by sexual behavior, sex, and nationality.

However, a clear trend was observed across all periods: the top 10% of viral load showed a progressive, although marginally non-significant (test for trend in proportions, *p* = 0.089), increase in the proportion of female individuals, rising from 27 to 35%, and a highly significant increase (test for trend in proportions, *p* < 0.001) in non-Italian nationals, rising from 5.3 to 18% (Table 1). In addition, when we analyzed the role of sexual behaviors, we observed a consistent predominance of heterosexual individuals among the top 10% of viral loads, with a gradual and significant increase in their proportion (test for trend in proportions, *p* = 0.011) since the 2002–2007 period, alongside a slight decrease in the proportion of homosexual individuals (Table 1). These trends were further supported by the evolving probability of belonging to the top 10% viral load group over time, with an intraclass correlation coefficient (ICC) of 0.62, indicating moderate clustering by sex, nationality, and sexual behavior. The corresponding probability of belonging to the top 10% viral load group across the subgroups and time periods is depicted in Figure 1.

**Table 1:** Demographic characteristics and sexual behavior of the top 10% viral load group and the bottom 90% viral load group.

Participants	[1997,2002] *N* = 2,492	[2002, 2007] *N* = 3,409	[2007, 2012] *N* = 3,916	[2012, 2017] *N* = 4,164	[2017, 2022] *N* = 4,202
Top 10%	92 (3.7%)	150 (4.4%)	184 (4.7%)	111 (2.7%)	190 (4.5%)
Sexual behavior					
Heterosexual	27 (67.5%)	48 (58.6%)	82 (66.2%)	50 (70.4%)	91 (77.8%)
Homosexual	10 (25.0%)	28 (34.1%)	37 (29.8%)	17 (23.9%)	18 (15.4%)
Bisexual	3 (7.5%)	6 (7.3%)	5 (4.0%)	4 (5.6%)	8 (6.8%)
Unknown	52 (56.5%)	68 (45.3%)	60 (32.6%)	40 (36.0%)	73 (38.4%)
Sex					
F	25 (27.2%)	39 (26.0%)	68 (37.0%)	37 (33.3%)	66 (34.7%)
M	67 (72.8%)	111 (74.0%)	116 (63.0%)	74 (66.7%)	124 (65.3%)
Italian nationality					
No	10 (10.9%)	8 (5.3%)	23 (12.5%)	20 (18.0%)	35 (18.4%)
Yes	82 (89.1%)	142 (94.7%)	161 (87.5%)	91 (82.0%)	155 (81.6%)
Bottom 90%	2,400 (96.3%)	3,259 (95.6%)	3,732 (95.3%)	4,053 (97.3%)	4,012 (95.5%)
Sexual behavior					
Heterosexual	773 (72.7%)	1,183 (69.0%)	1,445 (67.4%)	1,720 (66.8%)	1,741 (64.6%)
Homosexual	236 (22.2%)	428 (25.0%)	556 (25.9%)	680 (26.3%)	743 (27.6%)
Bisexual	54 (5.1%)	104 (6.1%)	142 (6.6%)	178 (6.9%)	212 (7.8%)
Unknown	1,337 (55.7%)	1,544 (47.3%)	1,589 (42.6%)	1,475 (36.4%)	1,316 (32.8%)
Sex					
F	689 (28.7%)	929 (28.5%)	1,072 (28.7%)	1,170 (28.9%)	1,107 (27.6%)
M	1,711 (71.3%)	2,330 (71.5%)	2,660 (71.3%)	2,883 (71.1%)	2,905 (72.4%)
Italian nationality					
No	238 (9.9%)	412 (12.6%)	560 (15.0%)	648 (16.0%)	685 (17.1%)
Yes	2,158 (90.1%)	2,847 (87.4%)	3,172 (85.0%)	3,405 (84.0%)	3,327 (82.9%)
Unknown	4 (0, 2%)	0	0	0	0

The percentages were calculated based on individuals with available data.

### Community viral load

Overall, our data showed a progressive decrease in CVL over the years. In particular, we observed a linear reduction in community HIV viral load after 2002, with values declining from approximately 900 log₁₀ copies/mL to approximately 600 log₁₀ copies/mL by 2012.

**Figure 2:**
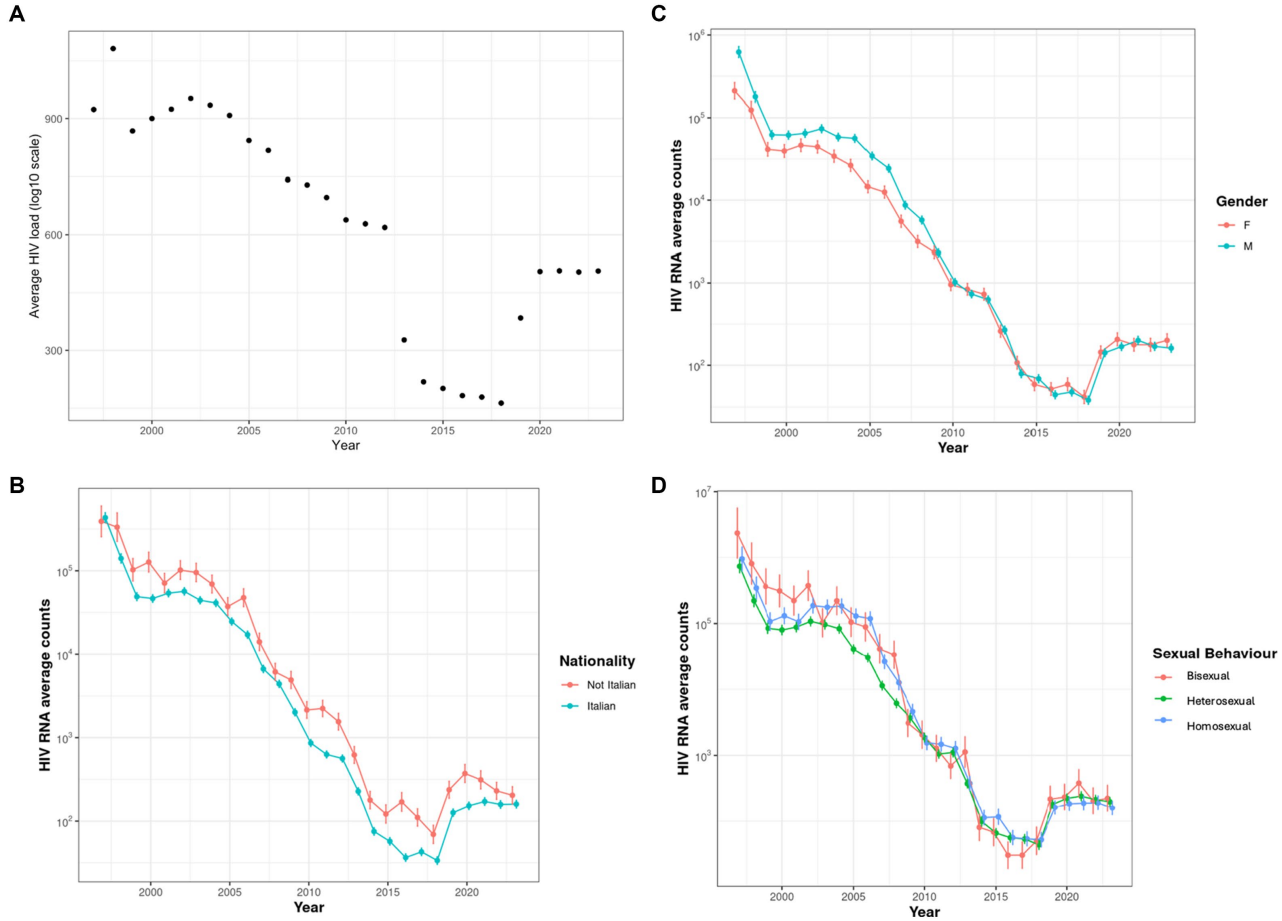
**(A)** Community viral load over time. **(B)** Community viral load categorized by nationality. Estimates (points) and 95% CI (vertical bars) of average HIV counts were obtained from GLMMs. **(C)** Community viral load categorized by biological sex. Estimates (points) and 95% CI (vertical bars) of average HIV counts were obtained from GLMMs. **(D)** Community viral load categorized by sexual behavior. Estimates (points) and 95% CI (vertical bars) of average HIV counts were obtained from GLMMs.

In 2012, we observed a sharp decline in community viral load to 150 log 10 cp/ml, followed by a continued gradual reduction. However, in the last 2 years, starting from 2020, a rebound in viral load was recorded in our population—probably in conjunction with the COVID-19 pandemic lockdown—with average values rising again to 500 log 10 cp/ml (Figure 2A).

When analyzing these trends by nationality, comparing Italian and non-Italian PLWH, we observed a similar overall pattern in both groups, with similar viral load values across the observed periods. Before 2000, there appeared a slight increase in viral loads in the Italian group, while from 2000 to 2006, the non-Italian population was observed to have higher values. After 2006, the two curves appeared more intertwined, with a clear predominance of higher viral loads in the non-Italian group (Figure 2B).

After dividing the population according to their biological sex, we observed a consistent increase in HIV viral load among male individuals, with rare and punctiform reversals of this trend observed in 2000, 2012, 2022, and 2023 (Figure 2C).

When we analyzed CVL according to sexual behaviors, dividing the population into heterosexual, homosexual, and bisexual groups, we found no clear or significant differences among the three groups. A slight tendency for higher average viral loads was observed in the homosexual group compared to the heterosexual group, although this pattern was frequently reversed (Figure 2D).

### The Gini index

Comparing the average viral loads and the Gini index over time, we observed a specular trend during the period, with stable homogeneity in viral load distribution across the population from 1997 to 2012, followed by an abrupt increase in disparity, from 0.1 to 0.6, after that year. Similar to CVL, 2020 also showed a trend variation, characterized by a new decrease in distributional inequality (Figure 3A). This trend was also observed when analyzing the Gini index by nationality, sex, and sexual behavior (Figures 3B–D).

**Figure:**
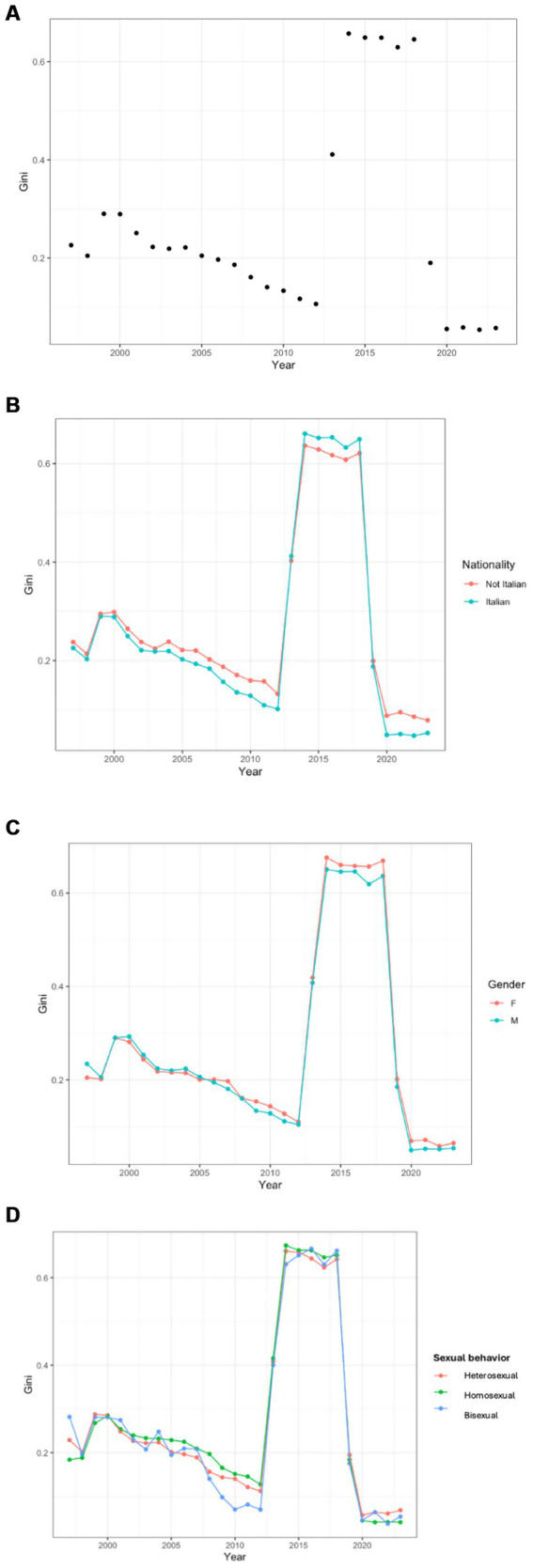
**(A)** Community Viral Load Gini Index over time. **(B)** Community Viral Load Gini Index over time, categorized by nationality. **(C)** Community Viral Load Gini Index over time, categorized by biological sex. **(D)** Community Viral Load Gini Index over time, categorized by sexual habit.

Considering nationality, we observed greater inequality among Italians compared to the non-Italian group during the period 2000–2012 and 2020–2022. In contrast, between 2012 and 2020, we recorded an opposite distribution, with higher homogeneity in the Italian group (Figure 3B).

When dividing by sex, we observed substantial parity in the homogeneity of the distribution from 1997 to 2012. After 2012, we observed a higher increase inequality among female individuals, which remained stable even during the decline in homogeneity recorded after 2020 (Figure 3C).

Analyzing sexual behavior, we observed a similar homogeneity pattern across the three groups, although the heterosexual group showed noticeably lower equality values from 2007 to 2015 (Figure 3D).

## Discussion

Since the advent of highly active antiretroviral therapy (HAART), the concept of treatment as prevention (TasP) has become increasingly central to limiting the spread of the virus within communities and controlling the global HIV epidemic (10, 11). Community viral load provides us with a key metric to assess the population’s HIV circulation and the potential impact of ART uptake on ongoing transmission. Previous studies have demonstrated a relationship between community-level viral load monitoring and HIV incidence, showing that increased ART coverage results in decreased viral loads and an overall reduction in the number of new HIV diagnoses each year (1, 2, 12, 13).

Our study measured community viral load at our clinical center over time and identified socio-demographic characteristics associated with higher viral load levels and increased transmission risk. We observed improvements in the HIV care cascade over the years, reflected by an overall decrease in community HIV viral load among PLWH at follow-up, consistent withprevious studies (14).

We observed a gradual decline in viral loads after 2002, uniformly distributed across the population. This could be due to the widespread use of therapies based on protease inhibitors introduced in the late 1990s, which had demonstrated clinical superiority over the earlier two-nucleoside combination regimens (15).

Furthermore, the introduction of integrase inhibitors (INSTI) appeared to have triggered a rapid declinein community HIV viral load in 2012, followed by a continuous gradual reduction. The INSTI class is arguably the most effective and well-tolerated ART developed to date and currently forms the base of the most recommended regimens for PLWH (16, 17). Moreover, by analyzing the trend of homogeneity in viral load distribution over time using the Gini index, we could observe the impact of the INSTI class (18) on the population. We observed substantial homogeneity in viral load distribution from 1997 to 2012, followed by an abrupt increase in disparity, from 0.1 to 0.6, after that year. This may indicate that while the majority of individuals achieved and maintained viral suppression, a smaller subset, potentially those with lower adherence, accounted for a disproportionately large share of the remaining viremia. In this context, the increased inequality in viral load distribution may paradoxically be a sign of overall therapeutic success, highlighting the central role of adherence in maximizing the benefits of potent antiretroviral therapy. Furthermore, innovative early treatment strategies, such as rapid initiation of therapy, have likely contributed to reducing community viremia by combining the rapid viral load suppression achieved with integrase inhibitors and maintaining viral response rates above 90% among people living with HIV (19).

In recent years, specifically during the 2 years following 2020, a rebound in viral load was observed in our population, likely coinciding with the COVID-19 pandemic lockdown. Previous studies have reported an initial decline in ART dispensation and a limited number of patients with a recurrence of detectable viremia, apparently due to the lockdown and restricted mobility during the pandemic (20–22).

The application of the Lorenz curve to evaluate the distribution of viremia showed a disproportionate viral load burden, with the majority of the virus being carried by 10% of the population, consistent with previous literature (6). We identified sex, nationality, and sexual behavior as significant risk factors associated with an increased likelihood of belonging to this top 10% viral load group. In our cohort, this group was consistently composed of male individuals, aligning with patterns reported in previous studies from North America (14, 23, 24).

However, other studies have reported an opposite trend, with a predominance of female individuals. In our cohort, we observed a progressive increase in the proportion of female individuals within the top 10% viral load group, rising from 29 to 35%, although the majority of the viral burden remained concentrated among men (1, 25, 26).

.A similar trend was also observed in non-Italian nationals, whose representation in the top 10% viral load group increased from 6.5 to 19%. This could be explained by known disparities in socioeconomic status and barriers to accessing prevention and care. These factors are often most pronounced in low-income subgroups, as demonstrated in the USA among African-American and Latino populations (1, 14).

However, we did not find a similar trend in the number of new HIV infection diagnoses, which remained stable at approximately 30%, highlighting the need to strengthen measures to improve compliance and retention in care within this population (27).

Calculating the Gini index, we observed greater inequality among Italian nationals compared to the non-Italian group during the periods 2000–2012 and 2020–2022, with the opposite pattern observed between 2012 and 2020. A similar trend was also evident when comparing male individuals to female individuals. This disparity may be influenced by both changes in the epidemiology of new infections and greater difficulties in achieving successful care cascade outcomes within these two subgroups.

When we analyzed the role of sexual behaviors, we observed a stable predominance of heterosexual individuals among the top 10% of viral loads, with a consistent gradual increase. This trend is not yet well established in the literature, with discordant data from North America: some studies confirm the predominance observed in our analysis (14, 25), while others report the opposite trend, showing a strong prevalence of men who have sex with men (24).

Similar to community viral load, in 2020, we observed a shift in trend, marked by a new decrease in the inequality of the distribution. This aligns with previous studies showing that only 1% of patients had viremia levels affected by the pandemic [22]-.

This study has several limitations. First, we did not consider some important social factors such as active injection drug use, housing situation, and engagement in sex work. Moreover, precise data on ART discontinuation and self-reported adherence would have been valuable for a more accurate interpretation of these analyses.

In our analysis, we did not exclude treatment-naive individuals, as the primary objective of applying the Lorenz curve was to identify and characterize those contributing disproportionately to community viral load, namely individuals with higher viremia and greater transmission potential. Treatment-naive individuals often fall into this category due to the lack of viral suppression, and excluding them would have limited our ability to fully capture the dynamics of population-level viremia and its distribution.

While we did not collect detailed data on treatment initiation timing for all individuals, we acknowledge as a limitation that the proportion of treatment-naive individuals likely varied over time, especially with changes in testing and treatment guidelines.

In addition, due to the heterogeneity and evolving nature of viral load monitoring intervals across the study period, shaped by changes in clinical practice, we were unable to provide a meaningful overall estimate (e.g., median or IQR) for the timing of viral load assessments.

Finally, CVL may underestimate the real burden of viremia as it excludes PLWH who are lost to follow-up and undiagnosed individuals. Efforts to improve retention in care and expand HIV testing coverage are crucial.

In summary, while advances in antiretroviral therapy have contributed to reductions in CVL and inequalities, effective HIV management requires a broader approach—one that addresses not only treatment efficacy but also socio-economic factors and equitable access to care. Public health policies that promote inclusivity and ensure access for all groups, regardless of nationality, sex, or sexual behavior, are essential for achieving more equitable management of HIV in the population.

Our findings suggest that focusing HIV prevention efforts on subgroups with the highest community viral load, in particular heterosexuals, female individuals, and non-Italian nationals, could produce the greatest reductions in the overall incidence of new HIV diagnosis. Addressing inequities in access to both care and prevention services is essential to reducing disparities in HIV incidence among subpopulations with the highest mean viral loads. Further identification of these individuals is needed to better target individual-level interventions.

Conversely, this is one of the few studies to apply both the Lorenz curve and the Gini index to community viral load, while also identifying subgroups at higher risk of an unsuccessful care cascade and with greater transmission potential. We demonstrated how important these tools are in characterizing HIV cohorts and assessing the relevance of social determinants in prevention and treatment strategies.

## Data Availability

The raw data supporting the conclusions of this article will be made available by the authors, without undue reservation.
